# Hospitalization and rehospitalization in Parkinson disease patients: Data from the National Parkinson Foundation Centers of Excellence

**DOI:** 10.1371/journal.pone.0180425

**Published:** 2017-07-06

**Authors:** Leili Shahgholi, Sol De Jesus, Samuel S. Wu, Qinglin Pei, Anhar Hassan, Melissa J. Armstrong, Daniel Martinez-Ramirez, Peter Schmidt, Michael S. Okun

**Affiliations:** 1 Department of Neurology, University of Florida Center for Movement Disorders and Neurorestoration, Gainesville, Florida, United States of America; 2 Department of Biostatistics, University of Florida, Gainesville, Florida, United States of America; 3 Department of Neurology, Mayo Clinic, Rochester, Minnesota, United States of America; 4 National Parkinson’s Foundation, Miami, Florida, United States of America; University of Pennsylvania Perelman School of Medicine, UNITED STATES

## Abstract

**Background:**

Patients with Parkinson disease (PD) are at high risk of hospital encounters with increasing morbidity and mortality. This study aimed to determine the rate of hospital encounters in a cohort followed over 5 years and to identify associated factors.

**Methods:**

We queried the data from the International Multicenter National Parkinson Foundation Quality Improvement study. Multivariate logistic regression with backward selection was performed to identify factors associated with hospital encounter prior to baseline visit. Kaplan-Meier estimates were obtained and Cox regression performed on time to hospital encounter after the baseline visit.

**Results:**

Of the 7,507 PD patients (mean age 66.5±9.9 years and disease duration 8.9±6.4 years at baseline visit), 1919 (25.6%) had a history of a hospital encounter prior to their baseline visit. Significant factors associated with a history of a hospital encounter prior to baseline included race (white race: OR 0.49), utilization of physical therapy (OR 1.47), history of deep brain stimulation (OR 1.87), number of comorbidities (OR 1.30), caregiver strain (OR 1.17 per standard deviation), and the standardized Timed Up and Go Test (OR 1.21). Patients with a history of hospitalization prior to the baseline were more likely to have a re-hospitalization (HR1.67, P<0.0001) compared to those without a prior hospitalization. In addition, the time to hospital encounter from baseline was significantly associated with age and number of medications. In patients with a history of hospitalization prior to the baseline visit, time to a second hospital encounter was significantly associated with caregiver strain and number of comorbidities.

**Conclusion:**

Hospitalization and re-hospitalization were common in this cohort of people with PD. Our results suggest addressing caregiver burden, simplifying medications, and emphasizing primary and multidisciplinary care for comorbidities are potential avenues to explore for reducing hospitalization rates.

## Introduction

Patients with Parkinson’s disease (PD) are reported to have 1.44 times more hospital admissions when compared to age and sex-matched peers, [1, 2] and these admissions are associated with prolonged length-of-stay and increased morbidity and mortality [3–5]. During hospitalization, approximately 20% of patients’ experience worsening of parkinsonian symptoms; 44% never return to their pre-hospitalization functional status [6, 7]. In a prior analysis using the National Parkinson Foundation Quality Improvement Initiative (NPF-QII), hospital encounters were shown to occur in approximately 30% of patients with PD who were followed prospectively for two years [8]. Following a first hospital encounter, the rate of a second encounter increased to approximately 50% when patients were followed into a second year [8]. The aim of the current study was to evaluate hospital encounters using a five-year follow-up period. Additionally, we aimed to identify factors associated with hospital encounters.

## Methods

### Study design

The study protocol, the informed consents and all study related documents submitted to the local IRB/IEC for review and approved prior to study initiation separately by every site. To monitor the progress of the study at sites, an annual complete continuing review report submitted to the IRB/IEC for approval. PD participants were enrolled in the study after written informed consent. The NPF-QII is an international, multicenter prospective longitudinal clinical study that includes over 7,500 PD patients from 20 sites followed prospectively for up to 5 years. Data collection was initiated in 2010 and is ongoing. The methods for the NPF-QII study have been previously described [9]. PD participants enrolled in the study after written informed consent. The NPF-QII study collects an annual standardized patient questionnaire which includes self-reported hospital encounters in their regular visit. For the purposes of this study, a “hospital encounter” was defined as either an emergency room (ER) visit or a hospital admission. Hospital admissions include surgical (not related to deep brain stimulation—DBS), non-surgical, PD-related and/or non-PD-related reasons; PD- and non-PD reasons are not distinguished. The cohort previously reported in Hassan et al 2013 (n = 3,415) is part of the current study cohort (n = 7,507) [8] and we have included follow-up data up to 4.85 years (median 1.85 years).

Variables collected include demographics (age and sex), number and type of comorbidities (including diabetes, cardiovascular disease, rheumatologic disorders, chronic pulmonary disease and neurologic disorders), living situation (home, nursing home, other) and regular care partner, Certainty of Idiopathic PD Diagnosis, clinical variables (subtype of PD, presence of motor fluctuations, Hoehn and Yahr stage, disease duration), quality of life measurements using the Parkinson’s Disease Quality of Life Questionnaire (PDQ-39) [10], ability to stand unaided, number of medications before baseline visit, along with medications and other therapy including levodopa, dopamine agonist, MAO-B inhibitor, COMT inhibitor, amantadine, cognitive enhancers, stimulants, antipsychotics, antidepressants, anticholinergics, DBS, occupational therapy, speech therapy, exercise program, mental health use, and social worker/counseling, timed up and go test (TUG), [11, 12] and the Multidimensional Caregiver Strain Index (MCSI) [13]. A family member or caregiver who accompanied patients at the visit completed the MCSI questionnaires.

Comorbidities were categorized according to cardiovascular, cancer, respiratory, arthritis, diabetes, other neurological disorders and an “other” category which includes any chronic disease such as musculoskeletal pain, arthritis, and infectious disease. Data collection was performed at baseline (patient first visit), year 1(one year after the baseline), year 2, 3, 4 and five (two, three, four and five years respectively after the baseline). The TUG test was performed by asking patients to stand up from a chair with their regular footwear and mobility aid, walk 3 meters, turn around, walk back to the chair, and sit down. The TUG test score was standardized by adding 6 seconds to the outcome if a patient was unable to perform without an assistive device (e.g. used a cane or walker)[14]. The PDQ-39 is a patient-completed quality of life questionnaire with 8 discrete scales in mobility (10 items), activity of daily living (6 items), emotional wellbeing (6 items), stigma (4 items), social support (3 items), cognition (4 items), communication (3 items), and bodily discomfort (3 items). These were scored based on the patient’s experience during the past month [10]. The MCSI questionnaire is completed by the caregiver and assesses physical strain, social constraints, financial strain, time constraints, interpersonal strain, and elder demanding/manipulative behavior [13].

### Statistical analysis

Analyses were performed using version SAS 9.4. Baseline demographics and clinical characteristics were summarized using mean ± standard deviation for continuous variables and by counts/percent for categorical variables. The primary analyses using the outcomes of ER visits and hospital admissions were performed separately with similar results, so they were combined into a single “hospital encounter” outcome. Multivariate logistic regression with backward selection was performed to identify factors associated with the encounter prior to baseline visit. Factors included demographic, social, diagnosis, and clinical variables, along with dummy variables indicating each medication and other therapy. Adjusted odds ratios (ORs) and 95% confidence intervals were provided for each factor, comparing to a reference group for categorical variables or per standard deviation increase for continuous variables. Kaplan-Meier estimates were obtained and Cox regression performed on time to hospital encounter after the baseline visit to better understand risks over time. Potential predictors included the same variables as the logistic regression, plus a dummy variable indicating whether the patients had a hospital encounter prior to baseline. In addition, Cox regression was repeated when restricting the cohort to the subgroup of patients who had a hospital encounter prior to the baseline visit. In current analysis, we excluded subjects who had missing data in any of the factors included in the final selected model. All associations with p-value<0.05 were reported, while those with p<0.0015 were claimed to be statistically significant based on Bonferroni adjustment considering all the variables examined in each regression analysis.

## Results

Seven thousand five hundred and seven patients were enrolled in the NPF-QII at the time of this analysis. Patient demographics are reported in Table 1. Of the 7,507 PD patients, 2,827 (37.7%) of patients did not have a one-year follow-up (1,427 were enrolled for less than a year and 1,400 had withdrawn from the study). When compared to the rest of the cohort, subjects withdrawn from the study were older, more likely to be female and without a partner or relative as a caregiver, and more likely to be categorized as H&Y stage >2 and have rest tremor, longer disease duration, more comorbidities, a longer TUG, a worse PDQ-39 score and more likely to have a hospital encounter prior to baseline visit (p<0.001). Specifically, 31.6% of the 1400 subjects who had withdrawn had hospitalization prior to baseline visit compared to 24.2% for the other 6107 subjects (p<0.001).

**Table 1:** Demographics of the 7507 patients at baseline visit.

Variables		N = 7507
**Demographic Variables**		
Age at first onset of PD symptoms		58.3±11.4
Age at baseline visit		66.7±9.9
Sex	Male	4718 (62.8%)
Race	White	5655 (75.3%)
	African American	122 (1.6%)
	American Indian	46 (0.6%)
	Multiple	23 (0.3%)
	Pacific Islander	9 (0.1%)
	Missing	1519 (20.2%)
**Social Variables**		
Living Situation	At Home	7210 (96.1%)
	Skilled Care	222 (3.0%)
	Other	67 (0.9%)
Regular Care Partner	No	1107 (14.8%)
	Spouse/Partner	5685 (75.9%)
	Other Relative	402 (5.4%)
	Paid Caregiver	241 (3.2%)
	Other	55 (0.7%)
**Diagnosis Variables**		
Certainty of Idiopathic PD Diagnosis
	≥ 90%	6420 (86.3%)
Rest Tremor presence
	Yes	5188 (69.7%)
Motor Fluctuations
	Yes	3588 (48.1%)
Hoehn and Yahr Stage	1	804 (10.7%)
	2	3639 (48.5%)
	3	1917 (25.5%)
	4–5	568 (7.6%)
	Not Assessed	579 (7.7%)
**Clinical Conditions**		
Disease Duration		8.9±6.4
Number of comorbidities		1.8±1.4
Number of Medications Before Baseline Visit		2.3±1.2
PDQ39 Total Score		25.2±15.9
MCSI Index		19.0±16.4
Standardized TUG		-0.1±1.0

### Rate of a hospital encounter prior to the baseline visit and factors associated with hospitalization

Of the 7,507 patients, 1919 (25.6%) had a hospital encounter prior to their baseline visit. Multivariate logistic regression analysis revealed that significant factors (p < 0.0015) associated with a hospital encounters prior to the baseline visit included being white (OR 0.49, CI: 0.33, 0.73), utilization of physical therapy (OR 1.47, CI: 1.24, 1.74), history of deep brain stimulation (OR 1.87, CI: 1.33, 2.62), the number of comorbidities (OR 1.30, CI: 1.22, 1.38), the MCSI score (OR 1.17 per standard deviation), and the Standardized Timed Up and Go Test (OR 1.21, CI: 1.1, 1.32) (Table 2). There are 24 variables not associated with prior hospitalization, which included age at baseline visit, sex, living situation, regular care partner, certainty of PD diagnosis, rest tremor presence, motor fluctuations, Hoehn and Yahr stage, disease duration, PDQ -39 emotional well-being, PDQ-39 total score, number of medications before baseline visit, dopamine agonist, MAO-B inhibitor, COMT inhibitor, amantadine, cognitive enhancers, stimulants, antipsychotics, anticholinergics, occupational therapy, speech therapy, exercise program, and social worker/counseling.

**Table 2:** Logistic regression analysis results on factors associated with hospital encounter prior to baseline visit.

Selected Effect	Adjusted Odds Ratio	95% Confidence Interval	p-value
Race (ref = white)	0.49	[0.33, 0.73]	**<0.0001**
Levodopa use (ref = no)	1.69	[1.27, 2.26]	0.002
Antidepressant use (ref = no)	1.18	[1.00, 1.40]	0.048
Physical therapy use (ref = no)	1.47	[1.24, 1.74]	**<0.0001**
Mental health use (ref = no)	1.42	[1.10, 1.84]	0.028
Deep Brain Stimulation (ref = no)	1.87	[1.33, 2.62]	**0.001**
Number of comorbidities	1.30	[1.22, 1.38]	**<0.0001**
MCSI Index (per standard deviation)	1.17	[1.09, 1.26]	**<0.0001**
Standardized TUG	1.21	[1.10, 1.32]	**<0.0001**

In addition to the 9 significant factors listed in the table, 24 other variables were eliminated during backward selection: 2 demographic variables (age, sex), 2 social variables (Living Situation, Regular Care Partner), 4 diagnosis variables (Certainty of Idiopathic PD Diagnosis, Rest Tremor presence, Motor Fluctuations, Hoehn and Yahr Stage), and 16 clinical variables (Disease Duration, PDQ Emotional Well-being, PDQ-39 Total Score, Number of Medications Before Baseline Visit, along with 12 dummy variables indicating the following medication and other therapy: Dopamine Agonist, MAO-B Inhibitor, COMT Inhibitor, Amantadine, Cognitive Enhancers, Stimulants, Antipsychotic Medicines, Anticholinergic Medicines, Occupational Therapy, Speech Therapy, Exercise Program, Social worker/counseling).

### Rate of hospital encounters after the baseline visit and factors associated with hospitalization

**Fig 1:**
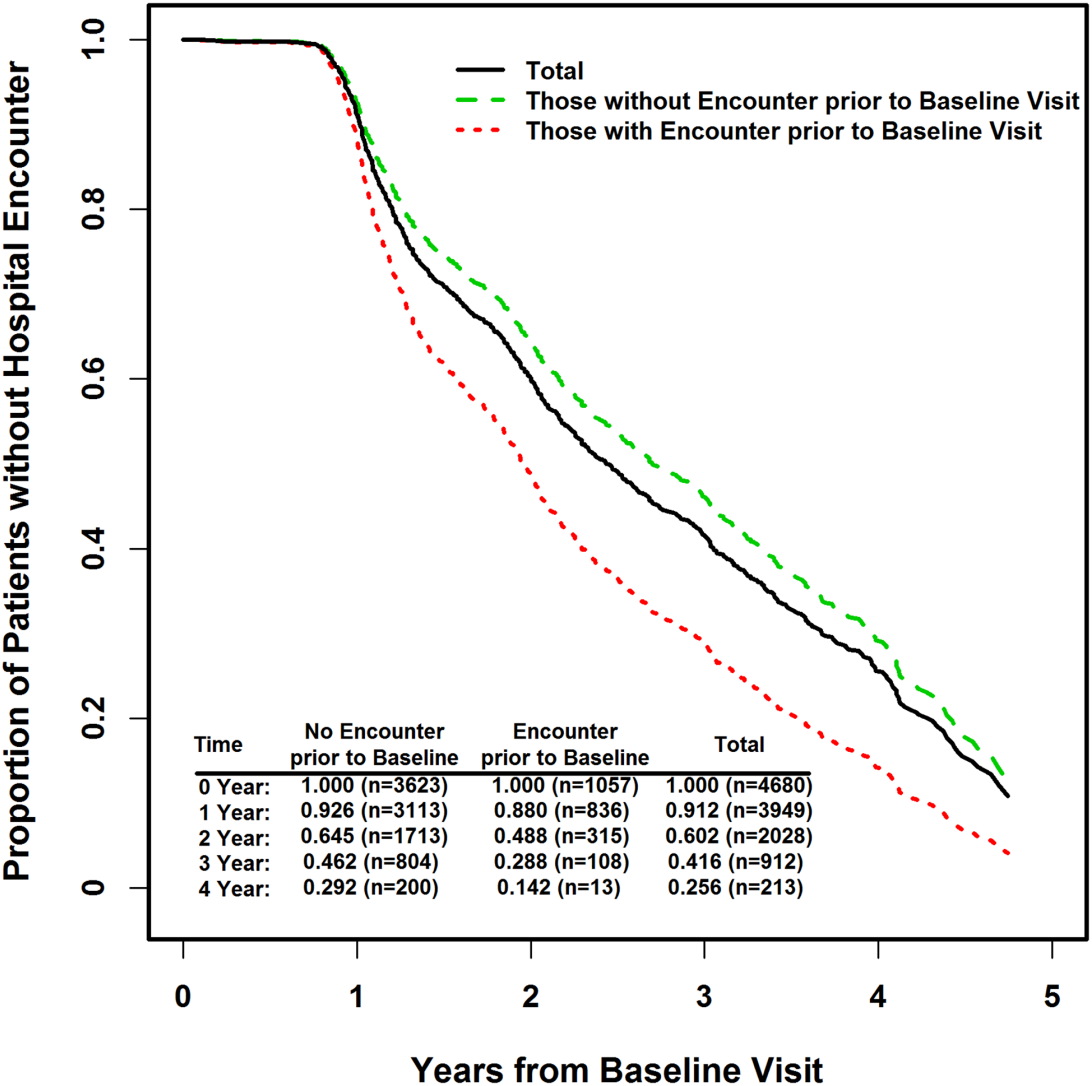
Estimates for proportion of patients without hospital encounter (i.e., 1 –rate of hospital encounter) over the years from the baseline visit. The results showed that, compared to those without hospital encounter prior to the baseline visit, those with encounter were significantly more likely to experience hospital encounter in the following years (Adjusted hazard ratio of 1.67, p<0.0001 from Cox regression). Estimated rates of ***no*** hospital encounter (and number of subjects at risk) were displayed at bottom-left corner of the plot by group and year.

Follow-up data was available for 4,680 participants. During an average follow-up of 2 years (median 1.85, maximum 4.85 years), 2,264 patients had a hospital encounter after the baseline visit. Compared to patients without a history of a hospital encounter prior to the baseline visit, those with a history of a hospital encounter were significantly more likely to experience additional hospital encounters in the following years (adjusted hazard ratio (AHR) 1.67, CI: 1.48, 1.89, p<0.0001 from Cox regression), as shown in Fig 1. Specifically, for PD participants with no hospital encounter prior to baseline, rates of ***no*** hospital encounter were 93%, 65%, 46% and 29% at the end of year 1 to year 4, respectively. This is in contrast to no (recurrent) hospitalization in 88%, 49%, 29%, and 14% for those patients with a hospital encounter prior to their baseline visit. The time to hospital encounter from baseline was significantly associated with age at baseline visit (AHR 1.17 per standard deviation of 9.9 years, CI: 1.10, 1.26), and number of medications (AHR1.10, CI: 1.05, 1.15) (Table 3).

**Table 3:** Factors associated with hospital encounter after the baseline visit estimated from Cox regression analysis.

	Adjusted Hazard Ratio	95% Confidence Interval	P-value
Age at baseline visit (per SD = 9.9 years)	1.17	1.10–1.26	**< .0001**
Number of comorbidities	1.06	1.01–1.10	0.0088
Number of Medications	1.10	1.05–1.15	**0.0001**
PDQ-39 total (per SD = 15.9 points)	1.11	1.04–1.19	0.0034
MCSI total (per SD = 16.4 points)	1.10	1.03–1.17	0.0057
Standardized TUG	1.09	1.02–1.17	0.0155
Levodopa (Yes vs No)	1.27	1.02–1.57	0.0302
Hospital Encounter prior to baseline visit (Yes vs No)	1.67	1.48–1.89	**< .0001**

### Frequent hospital encounters versus a single hospital encounter at follow up

Of the patients with a history of a hospital encounter prior to the baseline visit and at least one follow-up visit (n = 1057), 641 had one or more hospital re-encounters during follow up. This occurred an average of 1.6 ± 0.8 years (median 1.2 years) after the baseline visit. There were 416 patients who did not have a second hospital encounter during follow-up; differences between the cohorts are shown in Table 4. Time to a second hospital encounter was significantly associated with MCSI score (AHR 1.15 per standard deviation of 14 points, CI: 1.07, 1.26) and number of comorbidities (AHR 1.15, CI: 1.08, 1.24).

**Table 4:** Comparison of demographic, medication, treatments, and clinical characteristics between those with and without hospital re-encounter after the baseline visit.

Variable		Rehospitalization encounter after baseline visit		P-value*
		Yes (n = 641)	No (n = 416)	
**Demographic Variables**				
Age at baseline visit		67.8±9.7	66.5±9.6	0.032
**Diagnosis Variables**				
Motor Fluctuations	No	278 (43.4%)	220 (53.1%)	0.002
	Yes	362 (56.6%)	194 (46.9%)	
Hoehn and Yahr Stage	1	42 (6.6%)	56 (13.5%)	**<0.0001**
	2	248 (38.7%)	180 (43.3%)	
	3	229 (35.7%)	124 (29.8%)	
	4–5	76 (11.9%)	24 (5.8%)	
	Not Assessed	46 (7.2%)	32 (7.7%)	
Medications				
Levodopa	No	36 (5.6%)	48 (11.6%)	**0.0005**
	Yes	603 (94.4%)	366 (88.4%)	
MAO-B	No	506 (79.4%)	303 (73.7%)	0.031
	Yes	131 (20.6%)	108 (26.3%)	
COMT	No	481 (75.6%)	337 (81.8%)	0.019
	Yes	155 (24.4%)	75 (18.2%)	
Amantadine	No	484 (76.0%)	339 (82.5%)	0.012
	Yes	153 (24.0%)	72 (17.5%)	
Antidepressant	No	350 (54.9%)	282 (68.6%)	**<0.0001**
	Yes	287 (45.1%)	129 (31.4%)	
Other Treatment (after visit)				
Speech Therapy	No	549 (86.5%)	372 (90.5%)	0.048
	Yes	86 (13.5%)	39 (9.5%)	
Deep Brain Stimulation	No change	567 (91.7%)	384 (95.3%)	0.029
	Refer for eval	51 (8.3%)	19 (4.7%)	
First Year Clinical Condition				
Disease Duration		10.4±6.4	8.9±6.3	**0.0004**
Number of Comorbidities		2.4±1.4	2.0±1.3	**< .0001**
Number of Medications		2.7±1.2	2.3±1.2	**< .0001**
PDQ39 Emotion		7.1±5.1	6.2±4.7	**0.0015**
PDQ39 Total Score		29.7±16.0	25.2±14.7	**< .0001**
MCSI Index		22.7±16.4	18.9±15.0	0.003
Standardized TUG		0.2±1.0	0.0±1.0	**< .0001**

*P-values are from two-sample t-test for the continuous variables and a Chi-square test for the categorical variables.

## Discussion

In this 5-year longitudinal PD cohort with a mean disease duration of approximately 9 years at baseline, 25% had already experienced a hospitalization prior to baseline visit. Survival analysis revealed those with previous hospitalization compare to those without hospitalization had a significantly higher number of hospital encounters during follow-up. A history of hospitalization prior to the baseline visit was associated with race (white populations had a decreased odds of prior hospitalization), a history of receiving PT, a history of DBS, the number of comorbidities, the degree of caregiver burden, and the performance on the TUG. Prior hospitalization, age and number of medications were associated with subsequent hospitalization for the total cohort; on the other hand, caregiver burden and number of comorbidities were associated with a higher risk of re-hospitalization in those who already had a hospitalization at baseline. It is important to mention that many of the associations identified are likely to covary and might not represent independent associations.

The variables associated with a history of a hospitalization prior to the baseline visit are similar to those described in this cohort previously, and include longer TUG, higher number of comorbidities, the presence of motor fluctuations, and having DBS.[8] The increased risk of readmission in patients with a prior hospitalization is also confirmed in this updated analysis. This analysis builds on the prior work, however, this analysis followed participants over a longer duration of time and also further investigated factors associated with re-hospitalization. The factors associated with prior and new hospitalizations are not surprising, as many of these disease-related features indicate a more advanced disease state or the presence of other comorbidities. These findings are also consistent with limited prior literature on this topic. For example, reduced mobility and falls are common reasons for emergency hospital admissions in people with PD.[3]

These findings may provide new insights into mechanisms for preventing hospitalization in people with PD. Preventing hospitalization is critical where possible, as PD patients commonly experience a deterioration of symptoms during hospitalization and many do not return to their pre-hospitalization status [6, 7, 15, 16]. A recent systematic review, however, highlighted the lack of robust evidence for measures aiming to reduce hospitalization [17].

In this study the most relevant factors for hospitalization are age, medications and a prior hospitalization. The ideal approach would be to focus on simplifying medications. In those with a prior hospitalization, the focus then broadens to include caregiver burden, comorbidities and treatments. Based on our findings, potential mechanisms to explore for decreasing admissions and readmissions could include addressing caregiver burden and needs, simplifying medications, improving urgent care access for non-Caucasian patients, addressing balance, and emphasizing multidisciplinary care for comorbidities and close collaboration with primary care physicians.

Strengths of our study include the large sample size, the naturalistic longitudinal follow-up over five years, and the extensive clinical and demographic information gathered from PD Centers of Excellence. Limitations include lack of a control group, lack of details regarding the reason for hospital encounters, large attrition rates of subjects over the study period, small number of rehospitalized patients, and the idea that experiences at Centers of Excellence might not be generalizable to all patients with PD. The recorded data did not capture the reasons for losses to follow up, which could have included reasons that might have affected hospitalization rates including nursing home placement, insurance coverage gaps, the distance to visit a center, or death. For some of the associations (e.g. caregiver strain), it is not completely clear whether these are a risk factor for hospitalization or a result of hospitalization.

## Conclusions

Rates of hospitalization for patients with PD are high and increase when patients have experienced prior hospitalizations. Given known risks of irreversible clinical decline in patients with PD who are hospitalized, identifying strategies to prevent or decrease hospitalization rates for these patients is critical. Based on variables associated with hospitalization in this study, addressing caregiver burden and needs, simplifying medications, addressing balance impairments, and emphasizing primary and multidisciplinary care for comorbidities are potential routes to explore in future studies aimed at lowering hospitalization rates. Additional analysis of NPF center to center variation and practices may also shed light on potential best practices.
